# Segmental Testicular Infarction: A Case Report

**DOI:** 10.7759/cureus.26063

**Published:** 2022-06-18

**Authors:** Ali Almalki, Ibrahim Z Salloot, Mohammed Mahjoub, Rami Hasan

**Affiliations:** 1 Department of Urology, King Fahad Military Medical Complex, Dhahran, SAU; 2 Department of Urology, King Fahad University Hospital, Khobar, SAU

**Keywords:** testicular torsion, doppler sonography, scrotal pain, acute scrotum, segmental testicular infarction

## Abstract

Segmental testicular infarction is an uncommon condition; it is idiopathic in most cases and the pathophysiology behind it is unclear. Patients usually present with a sudden onset of testicular pain, which mimics conditions such as testicular torsion and epididymo-orchitis in clinical presentation and can sometimes be mistaken for a testicular tumor on ultrasound, which can mislead some urologists to opt for an unnecessary surgical treatment. However, with proper assessment and reassuring tests, surgical treatment can be avoided, and successful conservative management can be achieved.

## Introduction

Segmental testicular infarction is a rare condition that is challenging to diagnose. Its etiology and pathophysiology are unclear and most of the cases are idiopathic in origin. It most commonly affects patients between the second and fourth decades of life [1]. The clinical presentation can resemble testicular tumors, orchitis, or testicular torsion. Segmental testicular infarctions can be differentiated from testicular tumors with the help of high-frequency color Doppler ultrasound. This will help in reaching the proper diagnosis and successfully managing the cases conservatively.

## Case presentation

We report the case of a 50-year-old male without any known chronic medical illnesses. He was admitted under the care of general surgery as a case of acute appendicitis. On day one post appendectomy, the patient reported severe left testicular pain of one day's duration and denied any other symptoms. His vitals were stable and he was afebrile. Physical examination of the scrotum revealed tenderness of the left testis with no swelling or discoloration. Both testes had a normal position with intact cremasteric reflex. Investigations including complete blood count and biochemical and tumor markers (human chorionic gonadotropin, alpha-fetoprotein) were normal and urine analysis was also unremarkable. 

**Figure 1:**
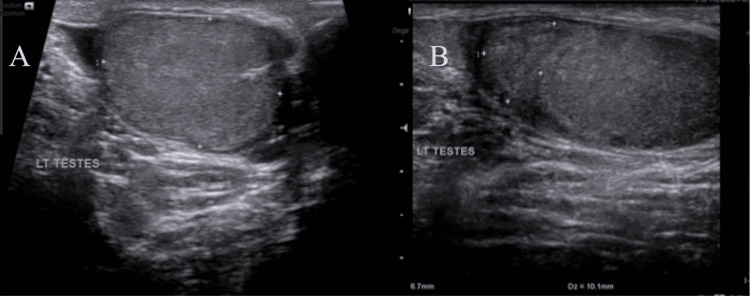
Initial testicular ultrasound of the left testis that revealed a homogeneous testis with an area of decreased parenchymal echogenicity

**Figure 2:**
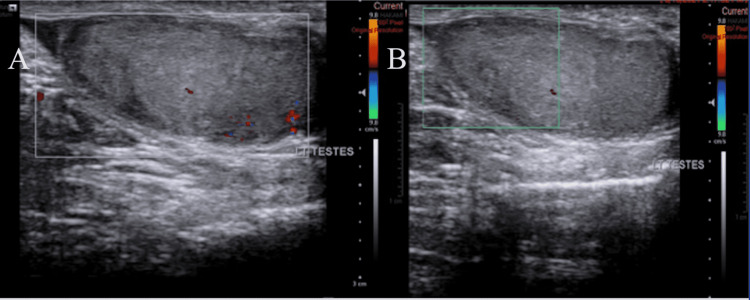
Initial color Doppler ultrasound of the left testis that showed a hypoechoic area with an absent color flow in that area

Scrotal color Doppler ultrasound was performed, which revealed an area of decreased parenchymal echogenicity in the left testis (Figures 1A, 1B) with an absent color flow in the area (Figures 2A, 2B).

**Figure 3:**
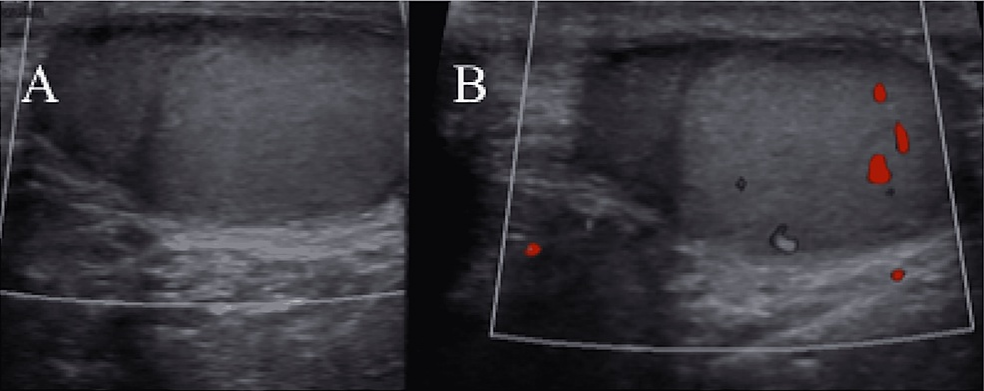
Follow-up color Doppler ultrasound that shows homogeneous left testis with a heterogeneous area with diminished blood flow related to remote insult

Based on ultrasound findings and laboratory test results, the patient was diagnosed with segmental testicular infarction. The pain completely subsided the next day after the patient was managed with analgesics. On follow-up a few months later, the patient was found to be asymptomatic. Re-evaluation with history and physical examination was unremarkable, and a repeat scrotal ultrasound demonstrated a heterogeneous area with diminished blood flow related to the remote insult (Figures 3A, 3B).

## Discussion

Segmental testicular infarction is a rare condition and around 70% of the cases are idiopathic. Epididymo-orchitis is considered to be the most common underlying cause of the condition [2]. Some identified risk factors include polycythemia [3], intimal fibroplasia of the spermatic artery [4], sickle cell disease [5], and trauma [3].

The patient, in this case, was initially admitted and managed as a case of acute appendicitis. However, the current literature shows no direct relationship between segmental testicular infarction and acute appendicitis or any other intra-abdominal infections. In theory, the testicles contain segmental areas that are considered functional end organs, and hence a reduction in blood flow due to various reasons such as venous thrombosis or arteriolar occlusion can lead to segmental testicular infarction [6].

The clinical presentation is nonspecific and might vary from one patient to another, but most cases present with acute scrotal pain, which was the presentation in this case. Clinical examination, testicular tumor markers, and imaging are important diagnostic tools. On clinical examination, the testicles are usually unremarkable but can be soft on palpation. Tumor markers are usually normal in cases of segmental testicular torsion, as shown in this case.

Ultrasound is the gold standard modality to evaluate any intra-testicular or extra-testicular abnormalities. B-mode ultrasound is sensitive in detecting any testicular lesions, but not accurate enough to differentiate between benign and malignant lesions [7]. Further assessment with color Doppler ultrasound for any increase or decrease in the blood flow may be needed to identify the nature of the lesion. In segmental testicular infarctions, a heterogeneous solitary solid wedge or a round area and the hypoechoic segmental area are usually seen with a significant decrease or absence of blood flow [5]. As Nayal et al. suggest, an avascular pattern in patients with no other evidence of malignancy would support the diagnosis of infarction [8]. With the color Doppler ultrasound findings and negativity of blood tests, especially with regard to tumor markers, the diagnosis of segmental testicular infarction can be suspected. Conservative management can be applied to these patients and invasive surgery can be avoided. Nevertheless, they usually need close follow-up to exclude malignancy [9].

Aquino et al. conducted a case series involving seven patients diagnosed with segmental testicular infarction [3]. The patient in the present case had a clinical presentation similar to most of the cases in the series and similar testicular ultrasound findings as well. In contrast to the patient in this case, who was successfully managed conservatively, all patients in the case series underwent orchiectomy.

## Conclusions

Segmental testicular infarction is an uncommon entity that mimics conditions such as testicular torsion, epididymo-orchitis, or a testicular tumor in clinical presentations. Conservative management is reasonable when color Doppler ultrasound findings are reassuring and testicular tumor markers are negative. By reaching the proper diagnosis and follow-up, unnecessary radical treatment can be avoided. In cases similar to the one described here, color Doppler sonography and blood tests are sufficient for the diagnosis and the watchful waiting approach is feasible and safe.
